# Effectiveness of 7-day triple therapy with half-dose clarithromycin for the eradication of *Helicobacter pylori* without the A2143G and A2142G point mutations of the 23S rRNA gene in a high clarithromycin resistance area

**DOI:** 10.3389/fmed.2023.1150396

**Published:** 2023-03-22

**Authors:** Seong Hyun Cho, Moon Sik Park, Seon-Young Park, Dong Hyun Kim, Hye-Su You, Hyun-Soo Kim

**Affiliations:** Division of Gastroenterology and Hepatology, Department of Internal Medicine, Chonnam National University Medical School, Gwangju, Republic of Korea

**Keywords:** *Helicobacter pylori*, eradication, clarithromycin, resistance, point mutation

## Abstract

**Background:**

Tailored therapy has been widely used for patients with *Helicobacter pylori* (*H. pylori*) infection in South Korea. Herein, we evaluated the treatment outcomes of tailored clarithromycin-based triple therapy (TT) in patients infected with *H. pylori*.

**Methods:**

We enrolled 460 patients without A2142G and A2143G point mutations by dual priming oligonucleotide-based polymerase chain reaction who had taken TT and undergone the urease breath test to evaluate eradication in clinical practice. Eradication rates according to the treatment duration and dose of clarithromycin were analyzed.

**Results:**

Among 460 patients (164 women, median age 63.0 years), 250 patients underwent TT with full-dose clarithromycin (TT-full CLA), and 216 patients underwent TT with half-dose clarithromycin (TT-half CLA). The eradication rates were 88.0% (220/250) in patients with TT-full CLA and 85.2% (179/210) in patients with TT-half CLA. In 250 patients with TT-full CLA, the eradication rates were 86.8% (33/38) in patients with 7-day TT-full CLA and 88.2% (187/212) in patients with 10-day or 14-day TT-full CLA (*P* = 0.788). In 210 patients with TT-half CLA, the eradication rates were 84.2% (139/165) in those with a 7-day TT-half CLA and 88.9% (40/45) in those with a 10-day or 14-day TT-half CLA (*P* = 0.436).

**Conclusion:**

For patients with *H. pylori* infection without A2142G and A2143G point mutations by DPO-PCR in clinical practice, treatment extension above 7-day TT with full CLA did not improve the eradication rates. Future studies on the treatment outcomes of TT-half CLA considering effectiveness and compliance are warranted.

## 1. Introduction

*Helicobacter pylori (H. pylori)* is associated with variable gastric diseases including peptic ulcer disease, gastric adenocarcinoma, gastric preneoplastic diseases, and gastric mucosa-associated lymphoid tissue lymphoma (1, 2). *H. pylori* eradication has been recommended for managing these potentially curable or related diseases (1, 3). However, the eradication success rate has decreased with the increase in *H. pylori* antibiotic resistance (4). Recent international guidelines such as the Maastricht V/Florence Consensus or Italian guidelines stated that other treatment regimens, rather than clarithromycin-based triple therapy (TT), should be considered in regions with high clarithromycin resistance in the absence of *H. pylori* susceptibility testing (5, 6). According to a recent nationwide survey conducted in South Korea, the *H. pylori* seroprevalence rate in 2015 and 2016 was 51.0% (7). Moreover, antibiotic resistance mapping of *H. pylori* in South Korea reported a clarithromycin resistance rate of 17.8% (4). Even though South Korea is now an area with high clarithromycin resistance (4), the Korean guidelines still state that clarithromycin-based triple therapy should be used as the first-line regimen for *H. pylori* eradication, which is specified to be used for 14 days (3). It also recommends performing a clarithromycin resistance test when 7-day clarithromycin-based TT is considered a first-line treatment (3).

There is an established association between clarithromycin resistance and various point mutations in the peptidyl transferase region domain V of the 23S rRNA gene (8). Recently, several molecular assays for detecting clarithromycin resistance in *H. pylori* based on the detection of mutations in the 23S rRNA genes have been suggested (1, 9, 10). Among molecular assays, a commercial dual-priming oligonucleotide (DPO) primer has been developed to detect single-nucleotide polymorphisms using a one-step PCR assay by Chun et al. (11). This test can be performed by gastric biopsy, even processed by the rapid urease test, and identify *H. pylori* with sensitivity, specificity, and concordance rates of 97.7, 83, and 90%, respectively, compared with bacterial cultures or the rapid urease tests (12–15). It can also verify the presence of mutations, especially A2142G and A2143G point mutations of the 23S rRNA gene related to clarithromycin resistance. There are several reports regarding *H. pylori* eradication rates of tailored TT in patients with clarithromycin-susceptible *H. pylori* determined by DPO-PCR. The overall eradication rate was 87–90% (16–19), which was close to those in TT without clarithromycin resistance determined by the culture method (20, 21). As bacterial *H. pylori* cultures and antimicrobial resistance tests are time-consuming, expensive, and difficult to perform, they cannot be employed easily in clinical practice. Conversely, molecular assays including DPO-PCR can be relatively easily accessible in clinical practice.

Even though tailored therapies based on the antimicrobial susceptibility test have improved eradication rates, eradication failure has still been reported in 10–15% of patients (16–19). Therefore, this study aimed to examine the efficacy of tailored TT considering treatment duration and the dosage of clarithromycin in patients infected with *H. pylori* without the A2143G and A2142G point mutations of the 23S rRNA gene in clinical practice.

## 2. Methods

This was a retrospective observational study. We identified 622 patients older than 18 years of age and younger than 80 years of age, in whom *H. pylori* infection was confirmed by a DPO-based multiplex PCR during esophagogastroduodenoscopy (EGD) and had undergone urease breath test for eradication verification after taking more than 80% of the prescribed medication in the Chonnam National University Hospital between Jan 2019 and July 2021. Among 622 patients, 151 patients with the A2142G and/or A2143G point mutations of the 23S rRNA gene were excluded from the study, as they received other treatment regimens other than TT. We also excluded 11 patients who did not take TT. Finally, 460 patients were included in this study. We extracted the data of demographics or clinical characteristics and eradication success rates according to the eradication regimens from the medical chart review. The Institutional Review Board of Chonnam National University Hospital approved this study (IRB Number: CNUH-2022-351). We followed the ethical principles of the Declaration of Helsinki.

### 2.1. Clarithromycin resistance test by PCR

We obtained two gastric biopsy specimens from the antrum and body of patients during EGD, for which a DPO-based multiplex PCR test (Seegen Inc., Seoul, Korea) was performed to diagnose *H. pylori* infection by detecting the deoxyribonucleic acid (DNA) extraction. The amplified DNA products were identified using an ultraviolet transilluminator in electrophoresis. A single 621-bp DNA product was considered a wild-type *H. pylori*. The presence of the A2142G and 2143G mutations resulted in DNA bands at 475-bp and 19-bp, respectively.

### 2.2. Eradication regimens and confirmation of *H. pylori* eradication

In this study, all patients were treated with acid suppressants [proton pump inhibitor (PPI)- or potassium-competitive acid blocker (P-CAB)]-based triple therapy, which consisted of 1000 mg of amoxicillin twice a day, 500 mg or 250 mg of clarithromycin twice a day, and acid suppressants [PPIs (rabeprazole 20 mg, pantoprazole 40 mg, oresomeprazole 20 mg twice a day), or P-CAB (50 mg of tegoprazan twice a day)] for 7, 10, or 14 days, respectively.

At least 4 weeks after treatment completion, the patients visited the hospital to take a standard ^13^C-urea breath test (UBiTKit, Otsuka Pharmaceutical Co. Ltd., Tokyo, Japan) to find out whether *H. pylori* eradication was successful or not. Eradication was defined as a negative result obtained in the test.

### 2.3. Statistical analysis

Continuous variables are expressed as the median and interquartile range (IQR), and categorical variables are expressed as numbers with percentages (%). Eradication rates according to the treatment duration (7 vs. 10 or 14 days) and dose of clarithromycin [full CLA (500 mg of clarithromycin twice a day) vs. half CLA (250 mg of clarithromycin twice a day)] were analyzed using Fisher's exact *t*-test. Two-tailed *P-*values of <0.05 were considered statistically significant. All statistical analyses were conducted using SPSS 27.0 for Windows (SPSS Inc., Chicago, IL, United States).

## 3. Results

### 3.1. Baseline demographic and clinical characteristics

We enrolled 460 patients without the A2142G and A2143G point mutations of 23S rRNA certified by the DPO-PCR test. Table 1 shows the baseline demographic and clinical characteristics of the enrolled patients. The median age (IQR) was 63.0 (57.0–70.0) years, and there were 164 (35.7%) women. There were 250 patients who had taken TT with full CLA (TT-full CLA) and 210 who had taken TT with half CLA (TT-half CLA). The most common indication for *H. pylori* eradication was gastric low-grade dysplasia (179, 38.9%), followed by carcinoma *in situ* or early gastric cancer (108, 23.5%). The overall eradication rates were 86.7% (399/460).

**Table 1:** Baseline demographic and clinical characteristics.

	**TT with full-dose clarithromycin (*n* = 250)**	**TT with half-dose clarithromycin (*n* = 210)**
Age, years, median (interquartile range)	62.0 (56.0–69.3)	64.0 (58.0–71.3)
**Gender**, ***n*** **(%)**		
Male	178 (68.8)	124 (59.0)
Female	78 (31.2)	86 (41.0)
**Body mass index, kg/m**^2^**, mean** ±**SD**		
Smoking, *n* (%)	31 (12.4)	23 (11.0)
Alcohol, *n* (%)	43 (17.2)	26 (12.4)
**Underlying diseases**, ***n*** **(%)**		
Hypertension	61 (24.4)	69 (32.9)
Diabetes	26 (10.4)	31 (14.8)
Chronic liver diseases	5 (2.0)	2 (1.0)
**Indication for eradication**, ***n*** **(%)**		
Carcinoma *in situ* or early gastric cancer	62 (24.8)	46 (21.9)
Low-grade dysplasia	101 (40.4)	78 (37.1)
Atrophic gastritis with/without intestinal metaplasia	24 (9.6)	29 (13.8)
Gastric polyp	26 (10.4)	25 (11.9)
Peptic ulcer	26 (10.4)	30 (14.3)
Others	11 (4.4)	2 (1.0)
**Duration of treatment**, ***n*** **(%)**		
7 days	38 (15.2)	165 (78.6)
10 or 14 days	212 (84.8)	45 (21.4)
**Types of acid suppressants**, ***n*** **(%)**		
Rabeprazole	19 (7.6)	0 (0.0)
Pantoprazole	170 (68.0)	77 (36.7)
Esomeprazole	26 (10.4)	25 (11.9)
Tegoprazan	35 (14.0)	108 (51.4)

TT, triple therapy; SD, standard deviation.

### 3.2. Eradication rates of *H. pylori* in patients who had taken TT with full-dose clarithromycin

Among the 250 patients who had received TT-full CLA, 212 patients received 10-day or 14-day TT-full CLA, and 38 patients received 7-day TT-full CLA. The overall eradication rates were 88.0% (220/250). There was no significant difference in the eradication rates of *H. pylori* in terms of treatment duration; 86.8% (33/38) in the 7-day TT-full CLA, and 88.2% (187/212) in the 10-day or 14-day TT full-CLA (*P* = 0.788).

### 3.3. Eradication rates of *H. pylori* in patients who had taken TT with half-dose clarithromycin

There were 210 patients who had received TT with half-dose clarithromycin (TT-half CLA). The overall eradication rates were 85.2% (179/210). Among them, 165 patients received TT 7-day TT-half CLA. The eradication rates of *H. pylori* were 84.2% (139/165) in the 7-day TT-half CLA and 88.9% (40/45) in the 10-day or 14-day TT-half CLA (*P* = 0.635).

### 3.4. Comparison of the eradication rates of *H. pylori* between patients with full-dose clarithromycin and those with half-dose clarithromycin

**Figure 1:**
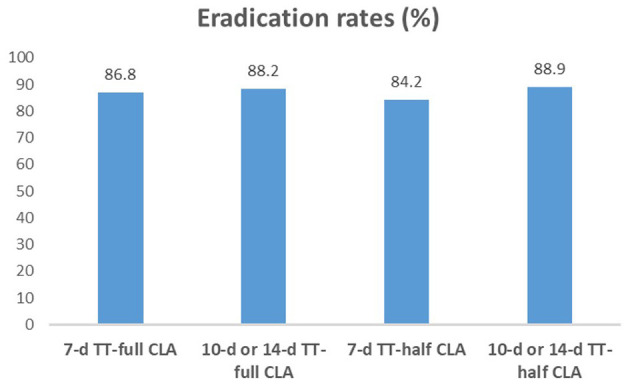
*H. pylori* eradication rates in each group. The eradication rates were not significantly different among the four groups (*P* > 0.05). In 7-day TT, there was no significant difference in the eradication rates between TT-full CLA (86.8%) and TT-half CLA (84.2%, *P* = 0.806). Moreover, in 10-day or 14-day TT, there was no significant difference in the eradication rates between TT-full CLA (88.2%) and TT-half CLA (88.9%, *P* > 0.999).

We compared the eradication rates among four groups (7-day TT-full CLA, 10-day or 14-day TT-full CLA, 7-day TT-half CLA, and 10-day or 14-day TT-half CLA). The eradication rates were not significantly different among the four groups (*P* = 0.806, Figure 1). We also performed the subgroup analysis according to the duration of TT. In 7-day TT, there was no significant difference in the eradication rates between TT-full CLA and TT-half CLA (*P* = 0.806). Moreover, in 10-day or 14-day TT, there was no significant difference in the eradication rates between TT-full CLA and TT-half CLA (*P* > 0.999).

## 4. Discussion

The efficacy of clarithromycin-containing regimens for *H. pylori* eradication has decreased due to increased antibiotic resistance (22–24). There are several alternative regimens to increase the efficacy of *H. pylori* eradication through the extension of the treatment duration and adjusting the antibiotic type and dose. These therapeutic regimens include bismuth quadruple therapy, sequential therapy, hybrid therapy, concomitant therapy, vonoprazan-containing triple therapies, or adjunctive therapies with probiotics (3, 5, 6, 25, 26). However, the use of these new regimens remains limited by the lack of efficacy improvement and gradually increasing adverse events. Another method of increasing the efficacy of *H. pylori* eradication is the antimicrobial susceptibility test-guided tailored treatment (19, 26). This study showed that the eradication rate of tailored TT-full CLA was almost 90%. There is no benefit in the extension of treatment duration of tailored TT-full CLA, and tailored TT-half CLA may be considered.

Clarithromycin resistance can be assessed by phenotype detection using a culture with the agar dilution method or the E-test and by genotypic methods using PCR. Even though culture methods can provide an overall evaluation of the clarithromycin resistance of *H. pylori*, regardless of the intrinsic mechanism involved, they are still time-consuming, and the cultivation of *H. pylori* is difficult even in expert hands (27). There are several molecular assays for detecting clarithromycin resistance in *H. pylori* based on the detection of mutation in the 23S rRNA gene (1, 8–10, 26). PCR-based techniques for *H. pylori* showed high sensitivity and specificity and provided the mutation types associated with antibiotic resistance. Several detection methods for point mutations at the 23S rRNA of *H. pylori* include PCR followed by restriction fragment length polymorphism analysis (10), quantitative PCR assay, DPO-based multiplex PCR, fluorescent *in situ* hybridization (28), or a peptide nucleotide acid probe-based qPCR test (9). Recently, multiplex real-time PCR has been used for one-step detection of the mutations of multiple sites to predict the resistance of clarithromycin and levofloxacin (29). In this study, a molecular method using DPO-PCR was used to identify key mutations (A2143G, A2142G) responsible for clarithromycin resistance. DPO-PCR had a sensitivity of 87.5% and a specificity of 91.3% on the basis of the C^13^-urea breath test (15). *H. pylori* without a point mutation certified by DPO-PCR has a low tendency for clarithromycin resistance. Although some guidelines recommend the 14-day treatment durations for *H. pylori* eradication (5, 25, 30), recent several studies showed acceptable eradication rates of ~90% with 7-day TT in patients with clarithromycin-susceptible *H. pylori* (31–33). A recent meta-analysis suggested that susceptibility-guided therapy was more effective than empirical therapy for treatment-naive patients, especially in areas where the clarithromycin resistance rate is >20% (34). In the present study, the eradication rates with 7-day TT-full CLA and 10-day or 14-day TT-full CLA against *H. pylori* without A2142G and A2143G point mutations were 86.8 and 88.2%, respectively, which were similar to the findings of previous studies (31–33). Therefore, TT beyond 7 days needs to be discussed considering various situations.

A previous meta-analysis demonstrated that half-dose clarithromycin-based therapy is equally effective as full-dose clarithromycin-based therapy for *H. pylori* eradication (35). However, clarithromycin resistance was not considered in that study. The dose of clarithromycin does not affect the therapeutic outcomes in the treatment of clarithromycin-resistant *H. pylori*. Therefore, to evaluate the pure effect of clarithromycin dosage (full-dose or half-dose) for *H. pylori* eradication, it is necessary to analyze the eradication rate in patients with clarithromycin-sensitive *H. pylori*. Clinicians need to choose antibiotic doses in terms of efficacy, antibiotics-related adverse events, and compliance. A large-scale observational study demonstrated that the adverse events increased with higher doses of clarithromycin (36). Therefore, half-dose clarithromycin may increase compliance by decreasing adverse events. In the present study, we compared the eradication rates of TT-half CLA against *H. pylori* without A2142G and A2143G point mutations. The eradication rate of 7-day TT-half CLA was 84.2%, which was regarded as unacceptable, while the eradication rates increased up to ~90% when the treatment duration was extended. Therefore, 7-day TT-half CLA may be insufficient even for clarithromycin-sensitive *H. pylori* eradication, especially in areas with high clarithromycin resistance. However, if eradication rates can be increased through more potent acid inhibition or treatment duration extension, it may be one of the treatment options with the benefit of decreasing adverse events and increasing compliance.

This study has several limitations. First, there is a lack of information about patients' compliance with regimens and adverse events, as this study was retrospective. However, we enrolled patients who took >80% of the prescribed medication, and there were no reported severe adverse events during the observational period of *H. pylori* eradication from the medical chart review. Second, there are the inherent risks of selection bias and information bias because this is a single-center retrospective study. Third, several types of acid suppressants (PPIs and P-CAB) were used for the eradication regimen. The metabolism of PPI is affected by the enzymatic activity, cytochrome P450 enzymes, and CYP2C19 with genetic differences. Conversely, P-CAB is not dependent on the CYP2C19 genotype and provides the long-standing inhibition of gastric acid secretion. While vonoprazan showed higher eradication rates compared with PPI (37, 38), tegoprazan used in this study showed similar eradication rates, compared with PPI (39). Moreover, there was no significant difference between PPIs and P-CAB for clarithromycin-susceptible *H. pylori* eradication (40). Therefore, the impact of the use of tegoprazan on the eradication rates may be insignificant in this study. Nonetheless, this is the first study showing the efficacy of TT-half CLA in patients with clarithromycin-susceptible *H. pylori*. In future, a randomized controlled study with a multicenter and large sample size needs to be performed to solve these problems.

In conclusion, in patients with *H. pylori* infection without A2142G and A2143G point mutations of the 23S rRNA, the extension of treatment for more than 7 day TT-full CLA did not improve the eradication rates. Future studies on the treatment outcomes of TT-half CLA need to be performed considering effectiveness and compliance.

## Data availability statement

The raw data supporting the conclusions of this article will be made available by the authors, without undue reservation.

## Ethics statement

The studies involving human participants were reviewed and approved by the Institutional Review Board of Chonnam National University Hospital (IRB Number: CNUH-2022-351). Written informed consent for participation was not required for this study in accordance with the national legislation and the institutional requirements.

## Author contributions

SHC and MSP contributed to the data collection and drafting of the manuscript. DK, H-SY, and H-SK participated in the review and editing of the draft. S-YP contributed to the study conceptualization, design, writing the original draft, and critical revision of the draft. All authors contributed to the article and approved the submitted version.
